# Structural, Mechanical, and Optical Properties of Laminate-Type Thin Film SWCNT/SiO_x_N_y_ Composites

**DOI:** 10.3390/nano14221806

**Published:** 2024-11-11

**Authors:** Elizaveta Shmagina, Maksim Antonov, Aarne Kasikov, Olga Volobujeva, Eldar M. Khabushev, Tanja Kallio, Sergei Bereznev

**Affiliations:** 1 Department of Materials and Environmental Technology, School of Engineering, Tallinn University of Technology, Ehitajate tee 5, 19086 Tallinn, Estonia; elizaveta.shmagina@taltech.ee (E.S.); olga.volobujeva@taltech.ee (O.V.); 2 Department of Mechanical & Industrial Engineering, School of Engineering, Tallinn University of Technology, Ehitajate tee 5, 19086 Tallinn, Estonia; maksim.antonov@taltech.ee; 3 Institute of Physics, University of Tartu, W. Ostwaldi 1, 50411 Tartu, Estonia; aarne.kasikov@ut.ee; 4 School of Chemical Engineering, Aalto University, Kemistintie 1, 02150 Espoo, Finland; eldar.khabushev@gmail.com (E.M.K.); tanja.kallio@aalto.fi (T.K.)

**Keywords:** barrier protective coating, carbon nanotube network, polysilazane, transparent optical thin films, nanocomposite coating

## Abstract

The development of new encapsulating coatings for flexible solar cells (SCs) can help address the complex problem of the short lifespan of these devices, as well as optimize the technological process of their production. In this study, new laminate-type protective composite coatings were prepared using a silicon oxynitride thin-film matrix obtained by curing the pre-ceramic polymer perhydropolysilazane (PHPS) through two low-temperature methods: (i) thermal annealing at 180 °C and (ii) exposure to UV radiation at wavelengths of 185 and 254 nm. Single-walled carbon nanotubes (SWCNTs) were used as fillers via dry transfer, facilitating their horizontal orientation within the matrix. The optical, adhesive, and structural properties of the matrix films and SiO_x_N_y_/SWCNT composite coatings, along with their long-term stability, were studied using Fourier transform infrared spectroscopy (FTIR), UV-Vis spectroscopy, HR-SEM, spectral ellipsometry, and a progressive-load scratch test. In this work, the optical constants of PHPS-derived films were systematically studied for the first time. An antireflection effect was observed in the composites revealing their two-component nature associated with (i) the refractive index of the SiO_x_N_y_ matrix film and (ii) the embedding of a SWCNT filler into the SiO_x_N_y_ matrix. The curing method of PHPS was shown to significantly affect the resulting properties of the films. In addition to being used as protective multifunctional coatings for SCs, both SiO_x_N_y_/SWCNT composites and SiO_x_N_y_ matrix films also function as broadband optical antireflective coatings. Furthermore, due to the very low friction coefficients observed in the mechanical tests, they show potential as scratch resistant coatings for mechanical applications.

## 1. Introduction

One of the key factors limiting the widespread use of flexible solar cells (SC) is their short lifespan [1]. This is primarily due to the degradation of the materials used inside the SC caused by oxygen, moisture, mechanical stress, temperature changes, and UV radiation [2]. The development of encapsulating barrier coatings, also known as protective coatings, is one possible way to overcome this complex problem [3,4,5]. By using different approaches to form such a coating, together with focusing on high barrier properties against moisture and gasses, it is possible to achieve a positive effect on the optical, mechanical, thermal, and other properties of the protective film [2]. In addition, such a strategy can reduce the number of functional layers involved, positively affecting the overall thickness and weight of the device, without the necessity of optimizing layer adhesion [3]. The creation of composite films is one prospective approach for enabling the integration of additional functional and protective properties into the framework of one single coating [6].

Various materials, such as epoxy resins [3], polymers [6], metallic glasses [7], etc., are being actively studied as protective coatings for flexible solar cells. When forming a composite coating, they can function as a base/matrix or as interlayers, depending on the architecture used [5,8]. Inorganic oxide and nitride films based on silicon are also actively studied barrier materials due to their long list of attractive properties [1], which are similar to those of glass, which is traditionally used for rigid SC protection [8]. These materials offer additional advantages, including the ability to create flexible, thin coatings that exhibit excellent adhesion to a wide range of materials, high resistance to temperature changes, and, in some cases, can be formed using low-temperature, vacuum-free processes [9,10,11]. These processes can be achieved by using a liquid pre-ceramic polymer from the extensive silazane family as a coating precursor. A prominent representative of this family is perhydropolysilazane (PHPS), which has recently attracted great interest from scientists due to its inorganic nature, allowing for the production of high-hardness barrier coatings [12,13]. The backbone of PHPS consists of a –Si–N–Si– chain with H atoms as substituents. [13]. It is compatible with liquid film formation methods, which are also scalable to industrial roll-to-roll processes, providing a substantial economic advantage [5,14].

Depending on the conditions of transformation or so-called curing (temperature and atmospheric composition), a deposited PHPS layer can be transformed into silicon dioxide, silicon nitride, and silicon oxynitrides (SiO_x_N_y_) of different compositions [8]. It should be noted that curing can even occur at room temperature [11]. The range of attractive properties of PHPS is illustrated by the growing variety of applications of this pre-ceramic polymer. PHPS-derived films have already proven their effectiveness as water, oxygen, and anti-UV barrier encapsulation layers for SC and microelectronic devices [15,16]. It has been demonstrated that PHPS-derived films can act very successfully as an anti-corrosion coating for low-carbon steel [17] and show promise as biodegradable materials for packaging applications due to their excellent barrier and optical properties [12]. More recently, PHPS-derived films have been shown to serve as a hydrogen permeation barrier, opening up new possibilities for their applicability in green energy technologies [18].

The creation of composites based on PHPS films can expand the range of functional properties of such coatings. For example, an optimally designed antireflective coating can significantly increase the efficiency of a SC. Due to reduced reflection, more light penetrates inside the device and participates in photoconversion [19]. This strategy has been demonstrated to work successfully for silicon dioxide thin films, increasing the efficiency of a perovskite solar cell by an additional 1% while having a positive effect on the self-cleaning properties of the coating [20]. A similar effect can be achieved for PHPS-derived films by manipulating the optical constants of the coating in various ways, including creating composites.

One of the disadvantages of PHPS-derived films is their high fragility, a consequence of the inorganic nature of the precursor. The creation of laminated composite coatings in which layers of PHPS alternate with organic polymer interlayers is a successful strategy for increasing the flexibility and reducing the brittleness of the protective coating, in addition to improving its barrier properties [5,8,21]. The unique properties of a composite coating are determined by the architecture and materials used. For example, PHPS-derived films have been successfully applied as interface layers in Cu/epoxy molding compound composites used in microelectronics. In these composites, PHPS-derived films increase the adhesion between the functional layers of the coating and improve barrier properties against moisture and ionic components [22].

The introduction of carbon nanotubes (CNTs) can potentially enhance the mechanical and thermal emission properties of the composite. It has long been discovered that CNTs have outstanding mechanical properties [23]. The addition of graphite and carbon black is traditionally used to increase the thermal conductivity of polymers, while CNTs have even more outstanding thermal conductivity values than other materials of the carbon family. Also, it was found that the thermal conductivity of single-walled carbon nanotubes (SWCNTs) is higher than that of multi-walled carbon nanotubes (MWCNTs) [24,25]. The use of CNTs as a reinforcing material is widely used in tandem with polymers and other matrices [26]. However, their application in PHPS-derived films has not yet been studied. The bulk properties of composites are highly dependent on their structure, so the problem of overcoming CNT aggregation has a strong impact on the development of such materials [25]. Alignment of CNTs inside the matrix, or in other words, the creation of a special architecture of the composite, can introduce required anisotropy of these properties [27].

In our previous study, we reported the successful formation of laminate-type composite thin films based on a PHPS-derived matrix with embedded SWCNTs as a filler [28]. During the initial characterization of the resulting SiO_x_N_y_/SWCNT composite structures, an antireflection effect was observed. To explain the effect, it is critically important to know the optical constants of PHPS-derived films cured by different methods. However, such data were not available in the literature, making it essential to obtain these measurements for the prepared SiO_x_N_y_ matrix films and their SiO_x_N_y_/SWCNT composites.

In this work, we characterized the optical properties of similar SiO_x_N_y_/SWCNT composite coatings and assessed their adhesion and behavior under progressive load tests. In this study, we close a gap in knowledge related to determination of the optical constants of PHPS-derived and cured films using various low-temperature approaches. The adhesive properties and behavior under the increasing mechanical load, as well as the long-term stability of the PHPS-derived films were also studied. The relationships between the chemical composition of the films and their properties immediately after curing and during the aging process of all the structures are reported for the first time. The antireflection effect observed in the SiO_x_N_y_/SWCNT composites was confirmed and associated with composite structure and curing of the coating. It was also determined that the SiO_x_N_y_ matrix films can independently function as antireflective optical protective coatings. The data obtained are particularly important for the practical application of the studied coatings in photovoltaic (PV) technology and other fields.

## 2. Materials and Methods

### 2.1. Samples Preparation

As a pre-ceramic precursor for SiO_x_N_y_ matrix layer formation, a commercially available 20% solution of perhydropolysilazane (PHPS) in di-butyl ether (NN-120-20, durXtreme GmbH, Darmstadt, Germany) was used. PHPS thin films were deposited by a Polos SPIN 150i spin-coater (SPS Netherlands, Putten, the Netherlands) at a speed of 2000 rpm for 60 s. As for the substrates, the soda-lime glass (SLG) and glass coated with a molybdenum (1 μm) (SLG/Mo) were applied. PHPS curing to SiO_x_N_y_ was performed using two low-temperature approaches: (i) thermally-induced (annealing at 180 °C for 60 min) in a Brother HD-14S oven (Zhengzhou Brother Furnace CO LTD, Zhengzhou City, China)and (ii) under UV-irradiation (185 + 254 nm for 40 min) in a Novascan digital UV ozone system (Novascan Technologies, Inc., Boone, IA, USA). Detailed information related to the PHPS films deposition, curing, and characterization can be found elsewhere [29].

Single-walled carbon nanotubes (SWCNTs) used as fillers were obtained via the aerosol chemical vapor deposition (CVD) with a floating catalyst using hydrocarbon feedstock (ethylene and toluene), ferrocene vapor as a catalyst precursor, and hydrogen as a carrier gas. The SWCNTs grown in the gas phase of a high temperature reactor have a mean bundle length of ~30 μm, a diameter in range of 1.4–2.3 nm and a density of approximately 0.12 g/cm^3^. These nanotubes were collected downstream of the reactor as thin films, oriented randomly in a horizontal plane on filter paper. Detailed information related to the filler properties is reported here [30,31,32].

SiO_x_N_y_/SWCNT composite films were formed in two ways: TOP and BOTTOM. In the first case, the SWCNT filler film was dry transferred from paper to a spin-coated and dried PHPS matrix film. In the second configuration, the SWCNT film was dry transferred to the substrate (SLG or SLG/Mo), followed by the deposition and drying of the PHPS layer. Both composite configurations were then cured. Treatment of the molybdenum surface with vapors from a 40% aqueous solution of HF acid for 10 min facilitated the dry transfer of the SWCNT film onto the Mo surface. More details related to the formation of SiO_x_N_y_/SWCNT composites and their primary characterization were reported in our previous study [28].

### 2.2. Characterization

A UV-Vis spectrophotometer (UV-1800, Shimadzu Corp., Kyoto, Japan) was used for the measurement of the transmittance and reflectance spectra of SLG-based samples in the 280–1100 nm wavelength range.

Spectral ellipsometry (SE) for samples on an SLG substrate was measured on the GES-5E (Semilab Co., Budapest, Hungary) device, equipped with the micro-spot option, in immersion mode with other glass sample. Propanol was used as the immersion fluid, and the angle of light incidence was set to 75°. Values of the reflection coefficients-ratio for different light polarizations (*tan Ψ*) and the phase difference (∆) of light reflected under different polarization were recorded and defined as:

(1) $$tan\Psi =\frac{\left|r_{p}\right|}{\left|r_{s}\right|}\;and\;\Delta =\delta_{p}-\delta_{s}$$

where $r_{p}$ and $r_{s}$ are Fresnel reflection coefficients for light polarized under p- and s-polarization states, and $\delta_{p}\;and\;\delta_{s}$ are phase components in p- and s-polarization states of the reflected light. The measured data were modeled and analyzed using SEA software in the energy range of 1.3–5.5 eV. The dispersion function was modeled using the Cauchy dispersion formulae:

(2) n=n0+Aλ2+Bλ4 and k=C+Dλ2

where *n* is the refractive index, *k* is the extinction coefficient (absorption index), and *λ* is the wavelength. *A, B, C*, and *D* are material coefficients. For modeling the glass substrates, the refractive index was taken as:

(3) n=1.535+0.0081λ2+0.00012λ4

based on former SE measurements.

For the Raman studies of SWCNTs, a Horiba LabRam HR800 (Horiba France SAS, Palaiseau, France) spectrometer in the backscattering configuration was used. The scattering was induced by a solid-state Nd:YAG laser (second harmonic, 532 nm) with a 5 μm spot size. The spectra represented in the article were recorded at a minimum of 4 different areas of the sample and then averaged to achieve more accurate results. Measurements were conducted in the 55–1700 cm^−1^ range.

The main method to study the degree of PHPS film transformation after the curing procedure is Fourier transform infrared spectroscopy (FTIR). For that, a Platinum-ATR Alpha (Bruker Optic GmbH, Ettlingen, Germany) spectrometer configurated with an attenuated total reflection (ATR) module was used. To exclude the influence of the glass signal on the measurement results, glass/Mo substrates were used. The measurements were performed in the range of 400–4000 cm^−1^ with a spatial resolution of 4 cm^−1^. For each sample, a minimum of 4 measurements were taken at different points. The nearly identical spectra were then averaged to obtain a representative result.

High-resolution scanning electron microscopy (HR-SEM, Zeiss Merlin, Oberkochen, Germany) was used to measure the thickness of coatings and study surface and cross-sectional morphology of the obtained layers. The accelerating voltage value was 4 kV. The samples were covered with a 1 nm Au (80%) and Pd (20%) coating using the magnetron sputtering technique to eliminate the low-conductivity effect.

To study the adhesion and behavior of the samples on Mo/glass substrates as the result of mechanical influence on coatings, a scratch test with a progressive load was performed using a universal materials tribo-test device, the CETR UMT-2 (Bruker, San Jose, CA, USA). For the tests, an HRC Rockwell spheroconical diamond indenter, with a radius of 100 μm and a cone angle of 120° was used. The surface of the indenter was cleaned with ethanol prior to each scratch. During the tests, the indenter moved along the surface of the films with a linearly-increasing load of 200 g/mm (1.96 N/mm), starting from 50 g (0.49 N) and ending at 2050 g (20.10 N). The length of each scratch was 10 mm. The scratching results were examined using HR-SEM. The critical load (Lc) was determined by the distance from the beginning of the scratch to the first visual appearance of cracks on the surface of the samples. The position of the indenter, applied load, and coefficient of friction (COF) were in situ monitored and recorded during the test to trace the changes in materials performance under the load.

## 3. Results and Discussion

### 3.1. Long-Term Stability of SiO_x_N_y_ Matrix Films

As described in the Materials and Methods section, the SiO_x_N_y_ matrix films were obtained by curing the pre-ceramic polymer PHPS using UV irradiation or thermal annealing. In our previous works, we reported that PHPS films cured by both these methods are not completely converted to silicon dioxide. Nitrogen is retained inside the films in different quantities, which was detected using FTIR, SEM energy dispersive X-ray (EDX) spectroscopy, and X-ray photoelectron spectroscopy (XPS). In addition, samples exhibited a tendency to age or ripen in a desiccator at room temperature, gradually replacing residual nitrogen with oxygen [29].

It should be noted that the long-term stability of the composition, structure, and properties of polysilazane-derived films, in general, has not been sufficiently studied. There is a lack of data on PHPS-derived films. Therefore, in this work, we conducted a detailed study of the dynamics of aging of matrix films using FTIR. The samples were aged in plastic boxes in a desiccator at room temperature without any additional influences. FTIR spectra for each sample were recorded in three marked areas and then averaged. To best track the dynamics of aging, spectra were recorded immediately after curing, after 24 h, after one week, three weeks, and six months.

**Figure 1:**
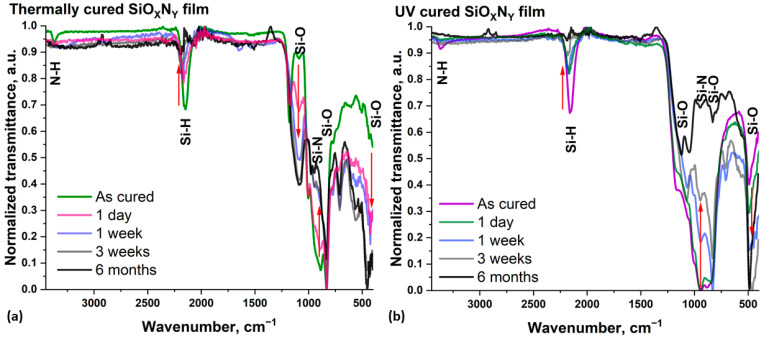
ATR-FTIR spectra of SiO_x_N_y_ matrix films measured during aging/ripening process of (**a**) thermally-cured sample, (**b**) UV-cured sample.

For both curing methods, as the samples age, the shape of the spectra underwent changes (the main changes in peak intensity are marked by red arrows in Figure 1a,b). In both cases, the samples had the same set of characteristic peaks, but their intensities differed, which led to spectra of different shapes.

The spectra of both freshly cured films reveal a band corresponding to the N–H bond stretching vibrations near 3365 cm^−1^ [33,34]. After a week of aging, this peak was no longer visible in either spectrum, or very broad, low-intensity vibration bands of the –OH group formed near this position up to 3000 cm^−1^ [14], likely due to the penetration of a small amount of water from the atmosphere into the film. This is also confirmed by the appearance of a small peak at about 1620 cm^−1^, caused by water molecules bending vibrations [35]. Around 2170 cm^−1^, bands corresponding to the stretching vibrations of Si–H bonds were observed [33]. The position of this peak shifted towards lower wavenumbers relative to standard values, which may indicate the presence of Si clusters inside the films [34]. During aging, the intensity of this peak noticeably decreased, almost disappearing after six months. Its position shifted slightly towards higher wavenumbers, which may indicate a decrease in silicon clusters and the formation of chemical bonds between silicon, and oxygen or hydroxyl groups as the samples age.

All other bands fall within the fingerprint region (1500 to 600 cm^−1^), where many different vibrational modes of silicon, nitrogen, hydrogen, and oxygen bonds are closely located, making their determination difficult. The band responsible for the stretching vibration of N–H in Si–NH–Si units is distinguishable in both spectra around 1180 cm^−1^ and is especially pronounced in the spectrum of the annealed sample [36,37]. As the films aged, the intensity of this peak decreased; after a week it was no longer clearly detectable for either curing method. Immediately after curing by both methods, the most intense band in the spectra was located around 954 cm^−1^ and was caused by Si–N stretching vibrations [38,39]. With aging, the intensity of this band decreased significantly. However, since at the same time vibration bands of the hydroxyl groups appear in the region of large wavenumbers, a low-intensity Si–OH stretching vibration may also emerge around 950 cm^−1^ [14]. In this regard, for aged samples we cannot correctly interpret the peak located in this position.

In the spectrum of a freshly thermally-cured sample, a low-intensity peak was detected at about 1089 cm^−1^, whose intensity gradually increased with aging, although its position remained unchanged. That is, the transverse optical (TO) mode of the Si–O–Si asymmetric stretching vibrations, and its position indicates the formation of clusters with a cage-like structure [36,40]. One week after UV curing, the peak representing this bond began to appear in the spectrum around 1068 cm^−1^. In the spectrum of a freshly cured sample, this band overlaps with neighboring bands, but as the sample ages, the relative intensity of this band increases, and its position shifts toward lower wavenumbers. After six months, this peak was located at 1050 cm^−1^, a peak position indicative of a quartz-like structure within the film [36].

In the spectrum of a six-month-aged UV film, a band around 1123 cm^−1^ appeared, which can also be attributed to the vibration modes of the Si–O–Si bonds [13]. The position of these peaks indicates that during the aging process, clusters with a cage-like structure and quartz-like structure are both formed inside the film. Bending vibrations of the Si–O–Si bonds are located around 830 cm^−1^ [14,41], but due to the change in intensity of the Si–H vibration peak located at 840 cm^−1^ it is difficult to analyze this region [42]. The rocking vibration of the Si–O–Si bond is localized around 490 cm^−1^ [8,43]. The intensity of this band increased most significantly during aging, eventually becoming the most prominent in the spectra of samples prepared using both curing methods.

In the case of a thermally-cured sample, the most noticeable changes in the shape of the spectrum occur during the first week after curing, then slowing down significantly. In contrast, for UV-cured samples, this process takes longer. As the films age, significant changes occur in their composition and structure. This process can be called ripening, where the oxygen content increases significantly, but the nitrogen and hydrogen content decreases. As a result, the peaks attributable to bonds between silicon and oxygen dominate in intensity, indicating that the composition of the films is close to silicon dioxide.

After six months of ripening, the spectra of films cured by different methods exhibit different shapes, reflecting differences in their structure, and, therefore, their properties. Notably, immediately after UV curing, the films contain less residual nitrogen and are closer in composition to silicon dioxide than thermally-cured films. This is evident from the FTIR spectra and is confirmed by the XPS and SEM-EDX data from our previous studies [28,29]. During the films’ aging, this trend continues, as confirmed by the SE results presented below. Therefore, if the goal is to obtain films that are close in composition to silicon dioxide, UV curing is the preferable method.

### 3.2. SiOxNy Composite Films with SWCNT

**Figure 2:**
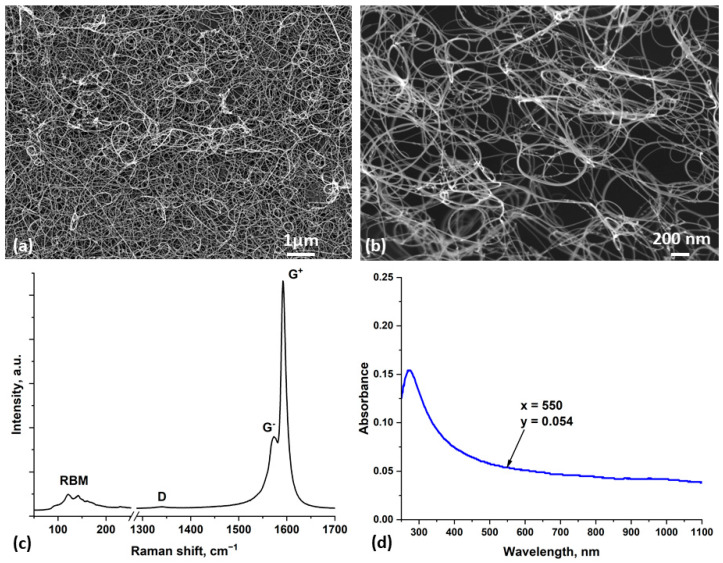
HR-SEM images of SWCNT film (**a**) onto SLG substrate and (**b**) onto carbon adhesive film, (**c**) Raman spectrum of a SWCNT film, and (**d**) SWCNT film on a glass substrate optical absorption spectrum with an absorbance of 0.054 at a wavelength of 550 nm (in the middle of visible wavelength range).

As described in the Materials and Methods section, SWCNTs were collected on filter paper to form thin films, with their thickness controlled by the deposition time. The papers with deposited SWCNT layers were then cut to produce pieces of any smaller size and shape. Figure 2a,b show HR-SEM images of the SWCNT film transferred by dry transfer to the surface of the SLG substrate and carbon adhesive tape, respectively. It is possible to determine the thickness of the SWCNT film using its optical absorption spectrum (Figure 2d). To calculate the thickness, the absorption value at a wavelength of 550 nm, which is 0.054, must be multiplied by the correlation coefficient value of 239 [44]. Thus, the SWCNT film used in this study had a thickness of approximately 13 nm.

The absorption spectrum contains only one peak at the wavelength of about 290 nm, caused by the absorption of π plasmons [45]. The absence of other absorption bands is due to the doping of SWCNTs by environmental species, such as oxygen, during storage of the films in the lab [46].

Figure 2c shows the Raman spectrum of a SWCNT film on the SLG substrate. The spectrum contains peaks of the radial breathing mode (RBM), clearly indicating the presence of single-walled carbon nanotubes. The very low intensity of the D peak around 1350 cm^−1^ indicates a small number of defects in the tubes. The splitting of the G peak at about 1591 cm^−1^ into two closely spaced components is also a sign of SWCNTs. The shape of this peak corresponds to high-quality nanotubes [47,48].

In this work, SWCNT films were used as a filler to obtain laminate-type nanocomposites in PHPS-derived thin film matrices. The composites were formed in two configurations: (i) BOTTOM, where the SWCNT film was transferred to the substrate surface before spin-coating the PHPS solution; and (ii) TOP, where SWCNTs were transferred to the surface of a formed, but uncured, PHPS film. Investigating these different configurations is important for controlling the distribution of SWCNTs within the matrix film, determining the possible influence of the configuration on the properties of the composite coating. Samples of both configurations were cured using both thermal and UV-assisted methods to thoroughly assess the possible change in their final properties.

**Figure 3:**
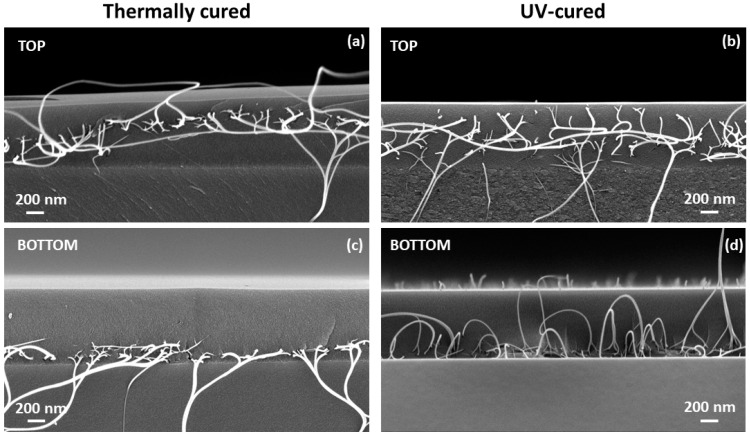
Cross-sectional HR-SEM images of SiO_x_N_y_/SWCNT composite films of the studied configurations (TOP and BOTTOM) on an SLG substrate, thermally-cured (**a**,**c**) or UV-cured (**b**,**d**).

Figure 3 shows cross-sectional images of cured SLG/SiO_x_N_y_/SWCNT composite samples in different configurations. For the BOTTOM configuration, the SWCNT film is located close to the SLG/SiO_x_N_y_ interface (Figure 3c,d). In the TOP configuration, SWCNTs are located predominantly in the upper layers of the SiO_x_N_y_ matrix film but may shift and spread deeper (Figure 3a). This behavior could be due to the diffusion of the stressed SWCNTs in viscous matrix material. During UV-induced curing, the film is cured gradually, starting from the surface downward, allowing the SWCNTs to remain longer in the viscous uncured matrix film, which can lead to their spreading throughout the volume of the matrix film (Figure 3b). When the sample is fractured to create a cross-section, the SWCNTs are stretched and torn. These fragments can be seen emerging from the film in the images. This illustrates the additional bonding effect of SWCNT fillers inside the composite film.

The process of formation of SiO_x_N_y_/SWCNT composites, along with their initial characterization, is described in more detail in our previous work [25]. In this study, we focused on studying the optical and mechanical properties of these composites.

### 3.3. Optical Constants and Properties of Prepared SiO_x_N_y_ and SiO_x_N_y_/SWCNT Coatings

Silicon-containing thin films with varying oxygen and nitrogen contents have been widely used as functional layers and coatings for optical and electronic devices for decades [34]. To successfully integrate such films into these kinds of applications, determining their optical parameters is critically important. While these data are well known for the boundary cases of silicon dioxide and silicon nitride obtained under different conditions [49], beyond their high optical transmittance, there are large gaps in information regarding the refractive indices (*n*) of PHPS-derived films cured by different methods, as well as the long-term stability of the optical properties of these films.

As was shown in our previous study, the degree of conversion of PHPS into SiO_x_N_y_ depends on the curing method used, which leads to the formation of films with different chemical compositions and structures [29]. The nitrogen and oxygen content within the film strongly influences its optical properties [50]. Additionally, FTIR results show that during aging/ripening, the SiO_x_N_y_ matrix films change their composition and structure. These changes are expected to affect the optical properties of the films.

There are various ways to determine the optical constants of films deposited on optically transparent substrates. These include spectroscopic ellipsometry (SE), which provides information about the refractive index, absorption index, and thickness of the film (*n*, *k*, d, respectively). SE detects the amplitude and phase of the light reflected from the sample for each wavelength of the range used. These quantities allow the optical constants to be calculated mathematically using suitable dispersion models. In our study, we used the Cauchy dispersion formulae to find the optical constants of the samples (Table 1). The convergence of theoretical data to experimental data were assessed using the R^2^ correlation parameter. The closer R^2^ is to 1, the closer the calculated and experimental data are.

**Figure 4:**
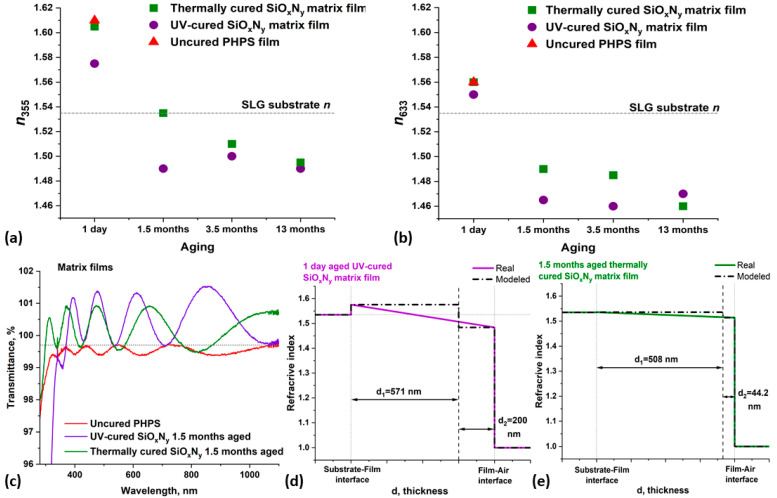
The refractive index values of the films as a function of aging time for *n* at wavelengths (**a**) 355 nm and (**b**) 633 nm. Transmittance spectra of uncured PHPS film and 1.5-month-aged UV and thermally-cured SiO_x_N_y_ films (**c**). The cross-sectional refractive index profiles of the SiO_x_N_y_ films for one-day UV-cured (**d**) and 1.5 months thermally-cured (**e**) SiO_x_N_y_ films.

Figure 4a,b show the dependences of the refractive index at wavelengths of 355 and 633 nm, respectively, on the aging/ripening time of the matrix films. To obtain the highest possible *n* value, an uncured PHPS film was prepared and measured, with the results shown in the figures as red triangles. This film contained the highest content of nitrogen and the lowest content of oxygen, which led to fairly high *n* values of about 1.6.

A thermally cured, one-day-aged SiO_x_N_y_ matrix film also exhibited *n* values very close to 1.6, in contrast to the UV-cured film, where *n* values are on average were 0.02 lower. In the UV-cured sample, the nitrogen content was lower in comparison with the thermally annealed PHPS film, which led to a decrease in the refractive index value. This is consistent with our previous results, illustrating the influence of the curing method on the structure and properties of the resulting SiO_x_N_y_ films.

In the UV-cured, one-day-aged SiO_x_N_y_ film, a fairly thick sub-layer in a two-layer model with d_2_ of 200 nm and an *n* value very close to the values for silicon dioxide, was formed on top of the d_1_ sub-layer, which has a higher refractive index (Figure 4d). The typical refractive index value for amorphous stoichiometric silicon dioxide is 1.465 at a wavelength of 633 nm [51].

The analysis of the transmittance spectra of samples makes it possible to improve the accuracy in calculating optical constants when modeling SE data. To achieve this, we measured the transmittance spectra of SiO_x_N_y_ matrix films cured by both methods and aged for 1.5 months (Figure 4c). All spectra were measured during a single working session of the spectrophotometer, and the uncoated SLG substrate was placed in the reference channel of the spectrophotometer. The uncured PHPS sample was used as a control, and its transmittance spectrum was measured two days after the film was formed. This sample exhibited the lowest transmittance of all measurements, indicating the highest refractive index, a value that exceeded the *n* value of the substrate.

Both cured samples had refractive indices lower than those of the substrate, which places their transmittance around the 99.7% level (our spectrophotometer channels are unbalanced by approximately 0.3%). The interference minima in the spectra of the cured samples are located below this level, as indicated by the gray line in Figure 4c. This indicates a negative inhomogeneity of the films, i.e., a gradient in the refractive index across the cross-section of the samples [52]. The beating seen in the spectrum of a UV-cured sample also points on to a possible inhomogeneity in the material [53]. Specifically, the *n* values at the substrate-film interface were higher than those at the film-air interface. The gradient was higher in the annealed film, as its minima are located lower relative to the gray line. Meanwhile, the UV-cured film had the lowest absolute value of *n* of all measured samples, since its spectrum is located above the others. All the observations described above are also reflected in the SE data (Table 1).

Based on Figure 4a,b, we claim that the refractive index value shows a tendency to decrease as the films age. This decrease is connected with a gradual reduction in nitrogen content and an increase in oxygen content in films cured by both methods, signaling their gradual ripening toward silicon dioxide. This is in good agreement with the FTIR results presented in Section 3.1. After a month of aging, the *n* values of the films approached the refractive index of the SLG substrate (gray dotted line in Figure 4a,b), or dropped below it, with further aging tending towards the *n* values characteristic for silicon dioxide.

As the samples ripen, the structure of sub-layers and the magnitude of the *n* gradient also change. According to the SE data, a one-day-aged UV-cured film had a rather thick upper sub-layer (¼ of the total thickness) with a smaller *n* value and a fairly large gradient value (Figure 4d). Previous reports have noted the formation of this top sub-layer, which is more converted to silicon dioxide than the buried sub-layer formed during UV curing [54]. In our previous work, we also detected this effect using the XPS and EDX line-scan techniques [29].

When a PHPS film was illuminated from the surface with UV light, the PHPS located closer to the film-air interface transformed more rapidly into a silicon dioxide sub-layer. This sub-layer begins to partially block the diffusion of oxygen deep into the film. As a result, the process of PHPS conversion in the underlying volume slows down. This creates a gradient of cross-sectional chemical composition in the UV-cured SiO_x_N_y_ film. Therefore, we do not see a step-like change in the composition and in the refractive index value, but rather a smooth transition, which is shown by the dash-dotted and solid lines in Figure 4d, respectively.

With aging, the magnitude of the gradient decreased so much that it was no longer noticeable in SE calculations (Table 1) and was only detected in the 1.5-month-aged sample using the transmittance spectrum (Figure 4c). In the case of thermal curing, the as-cured film was uniformly converted into silicon oxynitride, with a higher residual nitrogen content than the UV-cured PHPS film. However, after aging, the composition became similar to the UV-cured samples. An upper sub-layer with a lower *n* value compared to the rest of the material was detected (Figure 4e), which then ceased to be determined due to a decrease in the magnitude of the *n* gradient in the cross-section of the samples as they continued to ripen (Table 1).

We observed a decrease in both refractive and absorption index values for cured films as aging time increased. This change can be explained by the film composition shifting towards SiO_2_. The film thickness values are not representative due to the relatively non-uniform film thickness. The beam positions for SE measurements were not checked during the measurement. The SE data illustrates the general trends in changes in the optical values of films during their ripening, since the table represents data measured for different aging periods of the different films.

From the results of these measurements, it is clear that the ripening of UV-cured SiO_x_N_y_ matrix films proceeds noticeably faster compared to thermally cured samples. At the same time, UV-cured films exhibit refractive index values closer to the typical *n* values for silicon dioxide, due to a lower residual nitrogen content compared to annealed samples. This finding complements and expands the data obtained in this and our previous studies. The observed patterns were the same for the composite samples.

The results suggest that SiO_x_N_y_ matrix films can function as antireflective coatings for materials with refractive indices higher than theirs. As the SiO_x_N_y_ film ripens, the magnitude of the antireflection effect will increase due to an increase in ∆*n* between the film and the underlying material.

### 3.4. Antireflection Effect in SiO_x_N_y_ and SiO_x_N_y_/SWCNT Structures

In our previous work, we discovered an antireflection effect in a UV-cured SiO_x_N_y_/SWCNT composite sample in a BOTTOM configuration, which was confirmed by measuring the reflectance spectra [28]. In this work, we extended our measurements to a similar composite and the remaining samples studied in this work.

**Figure 5:**
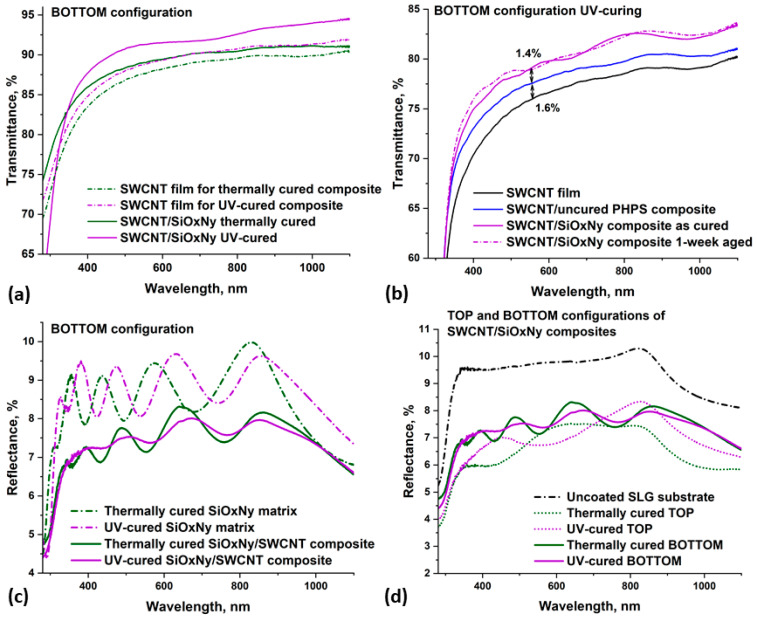
(**a**,**b**) transmittance spectra showing the antireflection effect in the SiO_x_N_y_/SWCNT composite samples; (**c**,**d**) reflection spectra confirming the presence of an antireflection effect in the SiO_x_N_y_/SWCNT composite samples.

In order to estimate the value of antireflection, the transmittance spectra of the SWCNT films transferred to the SLG substrate were first measured (Figure 5a). Then, in accordance with the BOTTOM configuration, PHPS/SWCNT composite films were prepared, which were cured by both thermal and UV assisted methods. As shown in Figure 5a, the transmittance of different areas of SWCNT film varied slightly, making it difficult to adequately assess the magnitude of the effect in TOP configuration composites. This was due to the inability to measure the transmittance of the specific piece of SWCNT film used in the formation of the TOP composite.

After curing, the transmittance spectra of the BOTTOM SiO_x_N_y_/SWCNT samples were measured again within the same operating session of the device, which eliminated the influence of additional factors associated with the operation of the spectrophotometer. For all spectra in this figure, measurements were taken vs. the uncoated SLG substrate in the reference channel. It can be seen that for both curing methods, the transmittance of the composite samples increased in comparison with the transmittance of a pure SWCNT film on the SLG substrate. In the case of UV curing, the increase in transmittance was higher (approximately 2.5%) than the thermally cured composite (approximately 1.4%).

For better evaluation of the observed effect, we studied the change in transmittance depending on the stage of formation of the composite film during UV curing (Figure 5b). First, the spectrum of the SWCNT film transferred to the SLG substrate was measured (black curve). A PHPS film was then spin-coated on top of the SWCNTs, and the spectrum of this uncured composite sample showed a 1.6% increase in transmittance in the mid-visible range (blue curve). After curing, the transmittance increased by another 1.4% (purple solid curve). All spectra were recorded within a single spectrophotometer run with the same baseline and without bare SLG substrate in the reference channel.

Thus, in the case of UV curing, we have two factors that influence the magnitude of the antireflection effect. The first is associated with the formation of the composite sublayer in the PHPS matrix (see Figure 3c,d) by the introduction of SWCNTs into the PHPS. The second factor, which increases the magnitude of the effect, is related to the the curing of the PHPS-derived matrix film accompanied by a decrease in the refractive index in the formed SiO_x_N_y_.

The increase in transmittance after the curing of PHPS is associated with a change in the refractive index of the coating during the curing process. The SE results showed that immediately after UV curing, the film had a fairly thick top d_2_ layer with a low *n* value, which led to the appearance of an antireflection effect. The broadband nature of the antireflection effect was caused by the presence of a gradient in the *n* across the film, which was identified in Section 3.3. Broadband antireflection is in great demand in many areas of photonics, as it replaces the construction of a traditional package of thin antireflection films with one coating, thereby simplifying and accelerating the manufacturing process [55,56]. This makes PHPS-derived matrix films very promising as antireflective coatings.

A week after curing, a new transmittance spectrum of the composite film was measured (dash-dotted curve). The transmittance value remained virtually unchanged after a week of aging, indicating that despite noticeable changes in the FTIR spectra, these changes are not sufficient to significantly affect the antireflective properties of the UV-cured coating.

An increase of 1.6% for the UV-cured sample, as well as an increase of 1.4% for the thermally annealed sample, are associated with the formation of the PHPS/SWCNT composite coating. The effect occurs when a SWCNT film is introduced into a matrix whose refractive index is higher than that of the SLG substrate. This phenomenon may be related to the creation of a nanostructured interface from the walls of SWCNTs within the PHPS matrix, which affects the propagation of light through the composite. Nanostructuring is one of the promising strategies for creating antireflection coatings [56].

Previous reports have highlighted a significant increase in transmittance (about 4.5%) and a decrease in reflection in nanostructured composite polymer electrodes with SWCNTs, especially when nanotubes are arranged as vertical arrays [57]. Additionally, the antireflection effect has been observed for SWCNT films randomly oriented in the horizontal plane deposited on the surface of a silicon wafer. With a small thickness (about 32 nm), the films were semi-transparent while acting as an antireflection coating for the substrate [55].

In the reflection spectra (Figure 5c,d), it is clear that both matrix films had less reflection compared to the SLG substrate. However, when SWCNTs were introduced into the matrix, the reflection of the composite film decreased even more, which confirms the presence of a synergistic antireflection effect.

It should be noted that the matrix films allowed an increase of around 1.4% in transmittance immediately after UV curing, which is a relatively small value compared to other developed coatings. On the basis of the results of SE, an increase in the transmittance value should occur as the value of *n* decreases with ripening for films cured by both methods. It is reported that by creating a microporous structure inside a silica film deposited on a glass slide substrate, it is possible to increase transmittance by just over 3%. This is caused by a decrease in the refractive index of the film due to the possible creation of a large number of pores [20]. Thus, one of the possible ways to increase the antireflection effect in PHPS-derived films can be connected with the controlled creation of inhomogeneous porosity in the deposited layer to decrease the *n* value closer to the film-air interface.

### 3.5. Progressive Load Test of SiO_x_N_y_ and SiO_x_N_y_/SWCNT Coatings

The scratch test is a standard method for assessing the mechanical resistance and adhesion of a coating to the underlying substrate. It helps to identify and study many different types of failures, such as coating peeling, plastic deformation, and film or substrate cracking. This test is conducted by pressing a special indenter onto the surface of the coating being evaluated and moving it with a constant or increasing load at a constant speed [58,59]. In our studies, we used a single pass of the indenter along the film surface under increasing load. This approach allowed us to determine the point of failure of the coating, which is called the critical load (Lc) point. Lc values were determined using HR-SEM by measuring the distance from the beginning of the scratch to the first visible damage to the coating. The initial load at the point of contact was 50 g, the rate of load increase was 200 g/mm, and the length of each scratch was 10 mm. In this study, we determine the Lc when the first visible damage occurred since the remaining modes of destruction were difficult to accurately determine.

During the tests, the change in the coefficient of friction (COF) of the films was also measured as the load increased. The coefficient of friction became unstable and began to increase sharply when the adhesion of the coating to the substrate changed (failure is initiated). Thus, the critical load can also be confirmed by measuring the change in the COF during the test.

The SiO_x_N_y_ matrix films and SiO_x_N_y_/SWCNT composite coatings were scratched one day, and one and three weeks after curing. It was found that mechanical behavior and adhesive properties were also critically dependent on the curing method.

**Figure 6:**
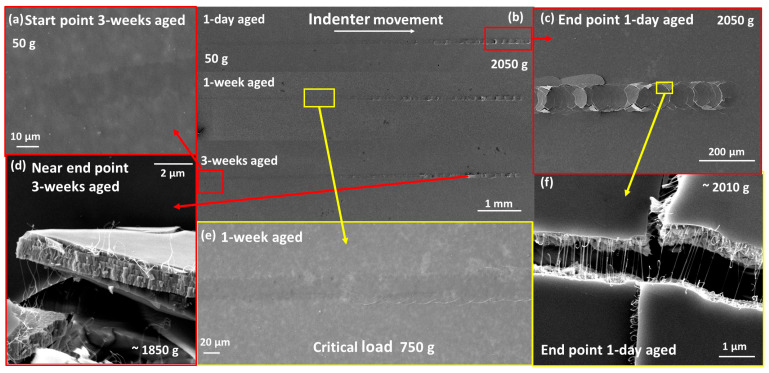
HR-SEM images of scratches from progressive load tests taken after one day and one and three weeks of aging of a UV-cured SiO_x_N_y_/SWCNT composite sample on a Mo/SLG substrate: (**a**) area of the indenter contact with the film, 50 g; (**b**) general appearance of all scratches; (**c**) the end of a scratch, 2050 g; (**d**) a fragment of the Mo/SLG substrate with a composite film on top of it; (**e**) the appearance of the first visible damage to the film, making it possible to determine Lc; (**f**) fragments of the substrate with a composite film on top of them, held together by stretched SWCNTs.

**Figure 7:**
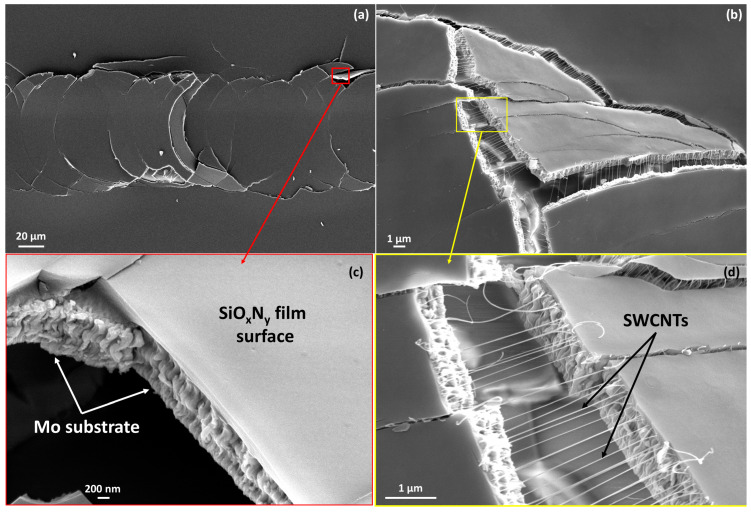
HR-SEM images of UV-cured samples: (**a**) a fracture of a scratch on a SiO_x_N_y_ matrix film under high load (near the end of the scratch) and (**c**) a magnified image of one of the fragments; (**b**) a scratch fragment on a SiO_x_N_y_/SWCNT composite sample and (**d**) its enlarged area.

UV-cured SiO_x_N_y_/SWCNT composites exhibited high hardness and excellent adhesion to the Mo/SLG substrate, maintaining these properties during the aging process (Figure 6b). The critical load value did not change noticeably with film aging/ripening. At low loads, no traces of the indenter remained on the samples (Figure 6a). The point of the indenter contact with the film surface can sometimes be determined only by the presence of a subtle contrast in SEM images or by measuring the length from the end of the scratch. After reaching the Lc, semicircular cracks began to appear on the surface of the sample. As the load increased, they turned into ring-shaped cracks. At high magnification, it becomes clear that fractures occurred in the substrate while the film remained tightly attached to the substrate fragments, thus illustrating very good adhesion to the molybdenum layer (Figure 6d and Figure 7c). This type of damage occurred due to the fact that brittle glass was located under the Mo layer. The compressive stress from the indenter passed deep into the film-substrate system and led to brittle fracturing of the glass [59]. Notably, the film did not peel off from the substrate beyond the scratch, although with very heavy loads, damage can spread beyond the scratch area.

The results for the UV-cured SiO_x_N_y_ matrix films are identical (Figure 7a,c). The introduction of SWCNTs into the matrix caused a visible reinforcing effect against rupture of the composite coating (Figure 6f and Figure 7b,d), however, the presence of one filler layer was not enough to influence the values of the critical load withstood by the coating.

**Figure 8:**
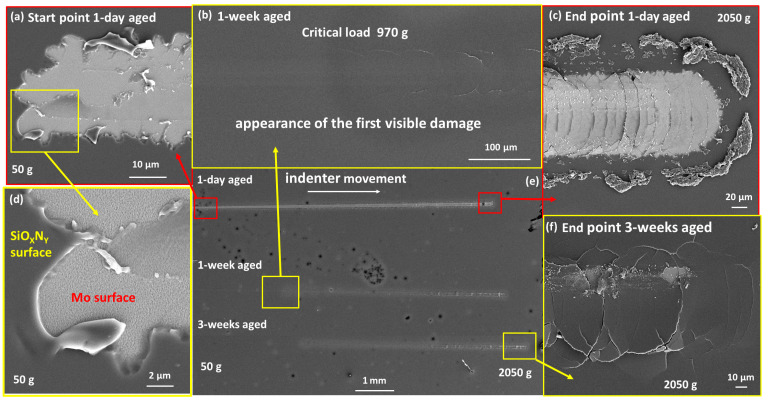
HR-SEM images of scratches obtained from the progressive load tests taken after one day and one and three weeks of aging of a thermally-cured SiO_x_N_y_ matrix film on a Mo/SLG substrate: (**a**) the contact point of the indenter with the surface, 50 g; (**b**) the appearance of the first visible damage to the film used to determine Lc; (**c**) the end of a scratch, 2050 g; (**d**) magnified image of the contact point, 50 g; (**e**) general appearance of all scratches; (**f**) end point, 2050 g. The aging time and load are indicated in the images.

**Figure 9:**
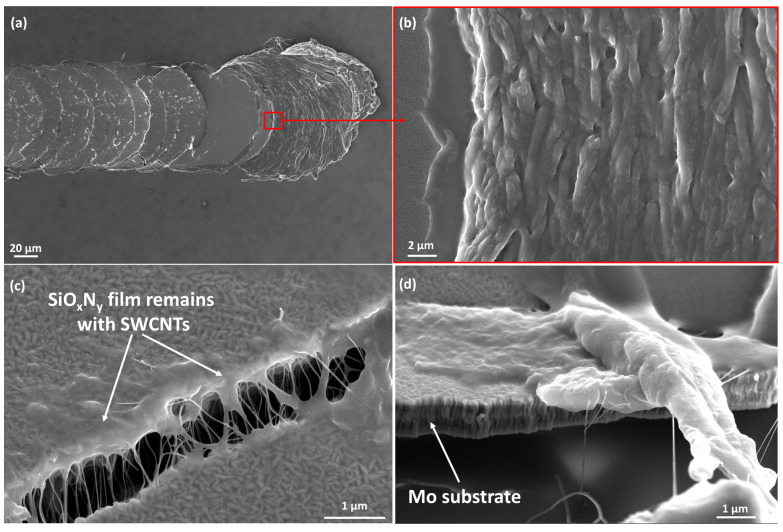
HR-SEM images of as-annealed SiO_x_N_y_/SWCNT composite sample: (**a**) destruction of the end of a scratch at 2050 g load and (**b**) magnified image of the plastically-deformed and extruded film beyond the end point of the scratch; (**c**,**d**) fragments of the substrate with the remains of the composite film inside the scratch track.

In contrast, all thermally-cured samples showed relatively lower adhesion to the Mo/SLG substrate, as well as lower hardness and high ductility (Figure 8a,c,d,e and Figure 9). Immediately after contact with the surface of the sample (at the lowest load of 50g), the indenter began to damage the coating (Figure 8a,d), with the scratch visually identified along its entire length (Figure 8e). The freshly cured film had very high ductility and was subject to a thinning effect. Under heavy loads, leaving behind only a very thin and uneven layer on top of Mo (Figure 9). Notably, the SWCNT film remained inside this very thin layer (Figure 9c,d). However, one week after curing, the behavior of the annealed films became similar to UV-cured samples, with adhesion to the substrate becoming strong and hardness increasing noticeably (Figure 8f). The Lc value increased several times, from 250 g for one-day-aged to 970 g for one-week-aged film. The results for the thermally cured SiO_x_N_y_/SWCNT composite film were similar.

This difference in the behavior of one-day-aged films is also associated with their cross-sectional structure after curing, which was studied in our previous works [28,29]. The plasticity and lack of hardness of fresh thermally-cured films are associated with the absence of a top layer of silicon dioxide and the presence of a very high nitrogen content throughout the film thickness. As the films age and ripen, the nitrogen content in the film decreases, and a layer similar in composition to silicon dioxide appears on the surface. These changes make the aged film more comparable to the UV-cured samples.

The value of Lc depends on many factors, including the thickness of the coating. The SE results indicate that the samples have different thicknesses, and the HR-SEM cross-sectional images showed some variation in thickness within the sample. Since we cannot always determine the thickness of the film over the entire scratch area precisely, it would be incorrect to compare the values of Lc without considering the thickness of the coating. Therefore, we do not present the Lc values of samples for all studied samples and scratches.

For all samples, the COF values did not exceed an average of 0.05. These values are an order of magnitude lower than those obtained by another scientific group during similar progressive load scratch tests for PHPS-derived films, but under completely different experimental conditions [17].

**Figure 10:**
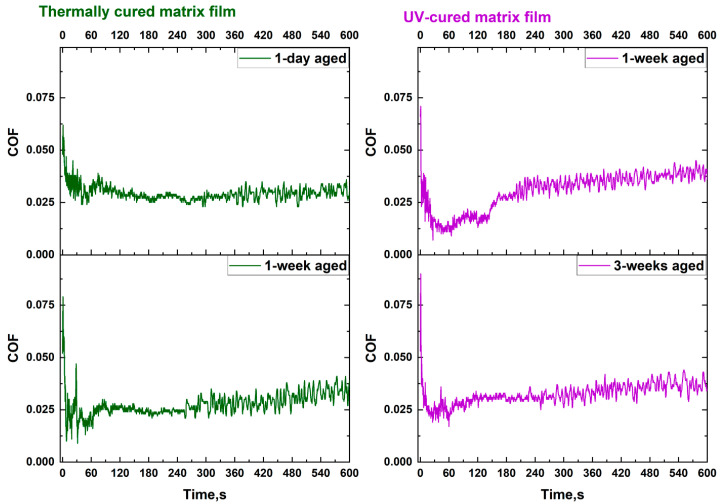
Typical friction coefficient curves recorded during progressive load scratch tests of SiO_x_N_y_ matrix films. Curing method and aging time are indicated in the pictures.

Such low COF values indicate the high smoothness of the film surface. Figure 10 shows coefficients of friction curves for some samples. For all samples, the same behavior was observed for the COF value depending on the scratching time. Several areas can be distinguished on the graphs. Initially, when the indenter made contact with the film surface (load close to 50 g), the COF had the highest values. This was followed by a significant decrease, with COF values sometimes approaching zero during the first 30 s. Then, there was a slight increase in COF, which then stabilized (with a very steady increase) as the indenter moved along the surface of the film.

Thus, the scratch test with progressive load showed that the composition of the films greatly influences the mechanical strength and adhesive properties. The introduction of SWCNTs into the SiO_x_N_y_ matrix did not affect the adhesive properties of the resulting composites. However, there was a visible reinforcing effect of the SWCNT network against the tearing of the SiO_x_N_y_/SWCNT coatings. Thermal curing has proven to be a less fortunate method for the formation of protective coatings from PHPS, while UV curing produces harder, more stable films with excellent adhesion to the Mo/SLG substrate. The low COF values make PHPS-derived matrix films and composites based on them promising coatings for improved lubricious and scratch-resistant properties, which is an especially important factor in many industrial processes [17,60].

## 4. Conclusions

In this work, the optical and adhesive properties of PHPS-derived SiO_x_N_y_ films cured by two low-temperature methods, as well as their long-term stability, were studied. Similar studies were conducted for laminate-type composite coatings prepared by introducing a SWCNT filler film into a SiO_x_N_y_ matrix film via dry transfer in different configurations. It was found that the PHPS-derived oxynitride films obtained by UV and thermal curing methods continue to ripen to silicon dioxide during the aging process. It was shown for the first time that active ripening during the first week after thermal curing leads to a change in the mechanical and adhesive behavior of the prepared films. The initial ductility and low adhesion are replaced by high strength and adhesion, similar to UV-cured films.

The incorporation of SWCNT fillers leads to an improvement of the resistance against rupture of the composite SiO_x_N_y_/SWCNT coatings. It has been confirmed that UV-cured films have a gradient in composition and refractive index in the cross-section, and thermally-cured films also acquire this feature during the ripening process. In this work, the optical constants of SiO_x_N_y_ PHPS-derived films, as well as their changes over time, were systematically studied for the first time. An antireflection effect was detected in the SiO_x_N_y_/SWCNT composite films, caused by the optical properties of the cured matrix film and the interaction of the matrix with the SWCNT filler. It was found that in addition to the presence of a refractive index gradient, when films age, their *n* value tends to approach the *n* values for silicon dioxide, which, coupled with excellent barrier properties, make these coatings a promising material for broadband antireflection and encapsulation of optics. Thus, the results of our study fill a lack of data related to the internal structure and properties of PHPS-derived films cured by low-temperature methods, as well as their long-term evolution. The low friction coefficients of the studied films can be useful to improve the sliding of mechanical parts and increase resistance to scratching.

## Figures and Tables

**Table 1:** Optical parameters of PHPS-derived films calculated on the basis of SE measurements.

Sample	Aging	d_1_, mm	*n*_355_	*n*_633_	*k*_355_	d_2_, mm	*n*_355_	*n*_633_	*k*_355_	R^2^
Uncured PHPS	1 day	673	1.61	1.56	0.08					0.99
Thermally-cured SiO_x_N_y_	1 day	625	1.60	1.56	0.09					0.99
	1.5 months	508	1.54	1.49	0	44.2	1.51	1.47	0	0.98
	3.5 months	598	1.51	1.49	0.06					0.99
	13 months	507	1.50	1.46	0					0.99
UV-cured SiO_x_N_y_	1 day	571	1.58	1.55	0.17	200	1.49	1.46	0.15	0.96
	1.5 months	752	1.49	1.47	0					0.99
	3.5 months	613	1.50	1.46	0.02					1
	13 months	598	1.49	1.47	0					0.98

## Data Availability

Data are contained within the article.
